# Understanding Scottish Dentists’ Attitudes and Knowledge Toward Dementia

**DOI:** 10.1002/alz70860_097725

**Published:** 2025-12-23

**Authors:** Freddie O'Donald, Molly Smith, Abigail Heffernan

**Affiliations:** ^1^ NHS Tayside, Dundee, United Kingdom

## Abstract

**Background:**

Dentists are well‐placed to observe changes in oral health that may indicate broader systemic conditions, including dementia. In Scotland, an ageing population has contributed to a rise in dementia cases, highlighting the importance of understanding dentists' attitudes and knowledge about dementia to support person‐centred care. While healthcare professionals’ preparedness for working with people with dementia has been studied, there is limited evidence focusing specifically on the dental profession. This study aimed to assess Scottish dentists’ baseline attitudes and knowledge related to dementia care.

**Method:**

A cross‐sectional survey was conducted with 56 dentists practising across Scotland, including both general dental practitioners and specialists. The Dementia Attitudes Scale (DAS‐6), a validated measure of person‐centred knowledge and comfort in providing care to people with dementia, was used alongside additional questions exploring training experiences, perceived confidence, and knowledge of dementia‐related behaviours. Data were analysed descriptively to identify trends and areas for improvement.

**Result:**

Survey respondents generally demonstrated positive attitudes toward dementia, with 92% recognising the importance of empathy and person‐centred care in supporting people with dementia. However, significant knowledge gaps were identified. Only 36% of respondents expressed confidence in identifying early cognitive decline, and 48% reported feeling prepared to manage oral health for people in advanced stages of dementia. Additionally, 64% of respondents were uncertain about effective strategies to promote self‐care in patients with dementia. Formal dementia care training had been completed by 78% of respondents. Open‐ended responses emphasised the need for practical guidance on recognising the early signs of dementia, tailoring treatment plans to meet the needs of patients at different stages of dementia, and enhancing communication strategies with patients and their caregivers.

**Conclusion:**

Scottish dentists showed positive attitudes toward dementia care but lacked confidence and practical knowledge in certain areas. Future research should examine the impact of targeted training on clinical practice and explore barriers to implementing dementia‐focused care. Limitations of this study include the relatively small sample size and the potential for response bias.

